# Functional validation of miRNA target genes in abiotic stress in Hippophae salicifolia

**DOI:** 10.6026/97320630018061

**Published:** 2022-01-31

**Authors:** Parneeta Chaudhary, Prakash Chand Sharma

**Affiliations:** 1 University School of Biotechnology, Guru Gobind Singh Indraprastha University, New Delhi, India

**Keywords:** Seabuckthorn, Hippophae salicifolia, miRNA, gene expression, abiotic stress

## Abstract

miRNAs are non-coding, single-stranded RNAs and are known to regulate the expression of genes during the post-transcription process. Seabuckthorn (Hippophae sp.; Elaeagnaceae) plant grows in different regions in harsh environmental conditions and is tolerant to various abiotic stress prevailing in the Indian Himalayas. Therefore, it is of interest to document the functional assignment of miRNA target genes to abiotic stress in Hippophae salicifolia using available bioinformatics tools. We identified eleven miRNA target genes in the seabuckthorn transcriptome. The expression analysis of these miRNA target genes provides important information about the regulation of stressresponsive defense mechanisms in seabuckthorn. Understanding of the role of these putative miRNAs and their target genes in cold and heat tolerance provides insights to determine the potential targets for the exploitation towards the development of stress-tolerant crop plants.

## Background:

Temperature stress is one of the most ubiquitous environmental factors that largely affect the growth and yield of crop plants [1]. The regulatory role of a large number of genes at the transcriptional level is known in regulating the response to temperature tolerance in higher plants [2]. The miRNAs have regulatory roles in facilitating the cleavage of mRNA [3,4], repression of translation [5,6], and negative regulation of the expression of genes at the post-transcriptional level [7,8]. The miRNAs have crucial roles in gene regulatory networks, multiple plant development mechanisms, and regulation of various metabolic pathways such as development [9-11], signal transduction [12], and response to abiotic [13,14], and biotic [15] stresses. To date, 38,589 miRNA sequences have been reported from 271 organisms, including plants, animals, and viruses, and are deposited in the public domain miRNA database (v.22.1) [16]. Seabuckthorn (Hippophae sp., family Elaegnaceae) has been recognized as an important medicinal and ecological plant since the ancient times and has recently gained the attraction of many researchers worldwide due to its multifarious nutritional properties. Therefore, it is of interest to identify the abiotic stress-responsive miRNA genes responsive to cold and heat tolerance in seabuckthorn (Hippophae salicifolia). The conserved miRNAs were identified among the various miRNAs via mRNAs from previously studied whole transcriptome assembly data of seabuckthorn [17], and expression analysis of genes during temperature stress was performed.

## Materials and methods:

### Sequence data:

The transcriptome assembly used in this study was developed in our laboratory and is already published [17]. A total of 10,410 reference mature miRNAs of viridiplantae taxa were downloaded from miRbase (miRBase Release 22.1), and a local database was created using OmicsBox (BioBam Bioinformatics, Spain)[18]. The redundant mature miRNA sequences were culled out manually and the remaining mature miRNAs were subjected to the pair-wise alignment against 88,297contigs of the assembled transcriptome [17] in subsequent analysis.

### Prediction of the potential miRNA targets:

The prediction of miRNAs targets was analyzed. psRNA Target server [19] was used with default parameters to predict the miRNA target. The potential miRNAs served as query searched against the mRNAs of seabuckthorn.

### Plant material:

The seabuckthorn (Hippophae salicifolia) plant saplings were collected from the High Altitude Medicinal Plants Seedlings Production Centre, Munsyari, Pithoragarh, Uttarakhand, India (Latitude: N30° 03.91; Longitude: E080°14.36; Altitude: 2182 m). The saplings planted in pots were maintained for further growth and used for total RNA isolation.

### Temperature stress treatment:

The plantlets were subjected to temperature regimes of 42°C and 4°C for time intervals of 2 hours, 4 hours, and 6 hours. The leaves were snap-frozen in liquid nitrogen and stored at -80°C until further downstream processing. The plantlets grown at 28°C were taken as control.

### Isolation of total RNA and cDNA synthesis:

The isolation of the total RNA from frozen seabuckthorn leaves was performed using the modified CTAB protocol [20]. The 1st strand of cDNA was synthesized using Qiagen 1st Strand cDNA Synthesis Kit according to the manufacturer's protocol.

### Primer design and relative gene expression analysis:

We utilized the freely available Primer3 (v.4.1.0) software to design the qRT-PCR primers [21] and checked through Gene runner (v.3.05) [22] software with complementation against these sequences for the validation of miRNA target genes during temperature stress. The actin gene, selected from an earlier publication, was considered for endogenous/housekeeping gene control [23], as the expression of actin is reported to be constant in most of the abiotic stress conditions. The list of eleven genes is summarized in Table 1(see PDF). To validate the expression of the miRNAs target genes, we further analyzed by relative quantification using 2-ΔΔCTmethod [24], of all the eleven genes. The fold change with the standard mean error was calculated using the standard deviation from technical triplicates and plotted as a bar graph to demonstrate the expression levels graphically. The complete methodology followed in the study is presented in Figure 1(see PDF).

## Results and Discussion:

Seabuckthorn transcriptome generated a total of 88,297 unigenes, which were further processed for the identification of miRNAs. In total, 10,410 mature miRNAs of viridiplantae taxa (miRBase Release 22.1) were downloaded from the public domain miRbase database [15] and were were taken as reference and searched against assembled transcriptome comprising of 88,297 unigenes, using locally installed OmicsBox (BioBam Bioinformatics, Spain) [25]. The resulted potential mature miRNAs showed homology with 682 unigenes and were subjected to BLASTx to remove the protein-coding region sequences among the potential miRNA candidate sequences. This exercise further led to the identification of ten miRNA sequences present in the transcriptome of seabuckthorn as summarized in Table 2(see PDF).

**Figure 2:**
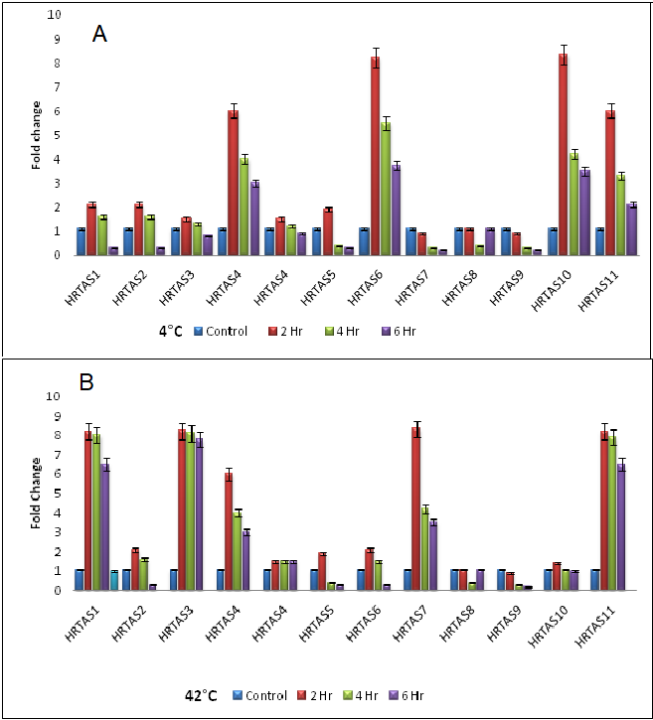
(A) Fold change calculated using qRT-PCR for the genes at 4°C, (B) Fold change calculated using qRT-PCR for the genes at 42°C.

The relative gene expression was determined by taking actin as the endogenous control (housekeeping gene) for the selected genes for various treatments. It was observed that out of eleven genes, only three genes i.e. HRTAS1 (NRG2P: nitrate regulatory gene2 protein), HRTAS3 (USPA: universal stress protein PHOS32-like), and HRTAS11 (unannotated gene) showed maximum unregulated expression at high temperature (42°C). The relative expression of genes HRTAS3 and HRTAS11largely reflects that the thermo-sensory mechanism process is activated at initial stages of stress treatment. However, after the exposure of 4 hour of stress duration, the treated sample showed dropped down level in expression. Such type of regulatory mechanism demonstrates the acclimatization of the plant to a specific stress condition. Considering the relative expression of the un-annotated target genes, HRTAS11, and HRTAS6, a variation in cold and heat conditions along with the time interval was documented. The early response to cold stress (4°C) and the gradual decrease of expression with respect to exposure (time interval) suggests the up-regulation of these unannotated target genes. However, the unannotated gene, HRTAS7, showed significantly high expression at high temperature (42°C) stress subjected plants. Among all four unannotated genes, which did not show any homology (taken for expression analysis) may be considered as unique and novel to seabuckthorn and the specific expression of HRTAS10 and HRTAS11 showed higher active regulatory mechanism at cold stress response and heat stress response, respectively. A study of these target genes confirms that the presence of these miRNAs and their target gene have a peculiar involvement in cold temperature-responsive genes and are specific to seabuckthorn genome. The fold change with the standard mean error, plotted as a bar graph to demonstrate the expression levels graphically has been shown in Figure 2A&2B.

Universal stress protein (USPA) gene is reported to show similar expression pattern in various plants, and are known as stress mediator that provide survival mechanism. In seabuckthorn, the NRG2P gene showed significant up-regulation at both the temperatures (42°C and 4°C). The contrasting expression level suggests that maximum expression is seen at high temperatures (42°C) as compared to cold temperatures. In the case of high temperature (42°C), a higher value of fold change suggests a large mediatory response. At low temperature (4°C), normal expression was perceived. Gene NRG2P has been reported with up-regulation, and this particularly is considered as a function of nitrate accumulation in plants. Moreover, responses to biotic stress, like anti-fungal, anti-parasitic protein mediatory action in Arabidopsis are also reported to be regulated by this gene [26].

## Conclusion:

We report putative miRNAs and their targeted genes that are known to play a significant role in various physiological and abiotic stress-related mechanisms, particularly in plants. The expression analysis of these target genes and their validation results by performing qRT-PCR assay has indicated the potential significance of miRNAs in the regulation of stress-responsive defence mechanisms in seabuckthorn. Data also shows their differential expression in response to cold and heat treatment and natural stress conditions in the environment. Exploitation of such miRNAs would help in understanding their role in cold and heat tolerance in seabuckthorn.
